# Telomere Dynamics and Homeostasis in a Transmissible Cancer

**DOI:** 10.1371/journal.pone.0044085

**Published:** 2012-08-29

**Authors:** Beata Ujvari, Anne-Maree Pearse, Robyn Taylor, Stephen Pyecroft, Cassandra Flanagan, Sara Gombert, Anthony T. Papenfuss, Thomas Madsen, Katherine Belov

**Affiliations:** 1 Faculty of Veterinary Sciences, University of Sydney, Sydney, Australia; 2 Devil Facial Tumour Project, Diagnostic Services, Animal Health Laboratory, Department of Primary Industries, Water and Environment, Launceston, Tasmania, Australia; 3 School of Biological Sciences, University of Wollongong, Wollongong, Australia; 4 Bioinformatics division, The Walter & Eliza Hall Institute of Medical Research, Parkville, Victoria, Australia; 5 Department of Mathematics and Statistics, The University of Melbourne, Melbourne, Victoria, Australia; University of Newcastle, United Kingdom

## Abstract

**Background:**

Devil Facial Tumour Disease (DFTD) is a unique clonal cancer that threatens the world's largest carnivorous marsupial, the Tasmanian devil (*Sarcophilus harrisii*) with extinction. This transmissible cancer is passed between individual devils by cell implantation during social interactions. The tumour arose in a Schwann cell of a single devil over 15 years ago and since then has expanded clonally, without showing signs of replicative senescence; in stark contrast to a somatic cell that displays a finite capacity for replication, known as the “Hayflick limit”.

**Methodology/Principal Findings:**

In the present study we investigate the role of telomere length, measured as Telomere Copy Number (TCN), and telomerase and shelterin gene expression, as well as telomerase activity in maintaining hyperproliferation of Devil Facial Tumour (DFT) cells. Our results show that DFT cells have short telomeres. DFTD TCN does not differ between geographic regions or between strains. However, TCN has increased over time. Unlimited cell proliferation is likely to have been achieved through the observed up-regulation of the catalytic subunit of telomerase (*TERT*) and concomitant activation of telomerase. Up-regulation of the central component of shelterin, the *TRF1*-intercating nuclear factor 2 (*TINF2*) provides DFT a mechanism for telomere length homeostasis. The higher expression of both *TERT* and *TINF2* may also protect DFT cells from genomic instability and enhance tumour proliferation.

**Conclusions/Significance:**

DFT cells appear to monitor and regulate the length of individual telomeres: i.e. shorter telomeres are elongated by up-regulation of telomerase-related genes; longer telomeres are protected from further elongation by members of the shelterin complex, which may explain the lack of spatial and strain variation in DFT telomere copy number. The observed longitudinal increase in gene expression in DFT tissue samples and telomerase activity in DFT cell lines might indicate a selection for more stable tumours with higher proliferative potential.

## Introduction

The world's largest carnivorous marsupial, the Tasmanian devil (*Sarcophilus harrisii*) has recently become threatened with extinction due to a unique transmissible cancer, Devil Facial Tumour Disease (DFTD) [1], [2], [3]. Prior to the emergence of the disease, devils were common throughout Tasmania. However, since the first sighting of DFTD in 1996, the disease has spread across the island state, resulting in population declines of up to 90% [3]. The disease now occurs in over 80% of the devil's geographic range, and the rapid population decline has led to the Tasmanian devil being listed as endangered by international (International Union for Conservation of Nature [4]) as well as national and state authorities [5].

DFTD is transmitted between individuals by biting during social interactions [6] and manifests in gross malignant tumours around the oral cavity, with frequent metastases to other organs [7], [8]. Due to starvation, secondary infections and organ failures, devils usually succumb to the disease within 6 months of tumour emergence [1], [7]. Cytogenetic analyses have revealed that DFTD is caused by a rogue clonal cell line [9], that is likely derived from cells of the neural crest lineage (Schwann cells) [8], [10]. Although DFTD possesses a highly rearranged genome, and is characterised by tumour specific complex translocations and chromosomal rearrangements, the cell line is remarkably (chromosomally) stable [9]. Recently however, four, closely related but karyotypically distinct DFT strains have been identified, suggesting that the tumour is evolving [11], [12]. Despite the four distinct DFT strains, genetic studies using microsatellite and immune-gene markers have demonstrated that Devil Facial Tumour (DFT) cells are genetically identical [10], [13], [14], [15].

Since their emergence in 1996 [9] DFT cells have undergone continuous division and propagation in thousands of devils without exhausting their capacity for replication and compromising their genomic stability. In contrast, normal human somatic cells display a finite capacity for replication, known as the “Hayflick limit”[16]. That is, after a given number of divisions, the cells exhaust their replicative potential due to shortened telomeres and enter a state known as replicative senescence. In hyperproliferative diseases, such as tumourigenesis, when telomere attrition reaches a critical level, cells enter a stage of growth arrest referred to as “crisis” [17], [18], [19]. By up-regulating or reactivating the telomere terminal transferase enzyme (telomerase), cancer cells are able to emerge from the “crisis” state and maintain telomeres that are slightly longer than those observed during “crisis” [17], [20].

Telomeres are ribonucleoprotein complexes at the ends of eukaryotic chromosomes essential in regulating cell lifespan [18]. Their primary functions are to ensure correct chromosome segregation during mitosis, and to prevent chromosome fusion and concomitant cell-cycle arrest, caused by the end of the chromosomes being treated as DNA double-strand breaks [21]. In vertebrates, including Tasmanian devils, telomeres consist of variable numbers of tandem repeats (TTAGGG nucleotides) bound by a specialised multiprotein complex known as shelterin [22], [23]. Telomere length homeostasis in germ line and tumour cells is achieved through the negative feedback loop of the shelterin complex and the telomere terminal transferase enzyme [24]. Telomerase is activated in pluripotent embryonic, and adult stem cells, to arrest the progressive telomere attrition through the addition of TTAGGG repeats to the 3′ strand of chromosomes [24]. Most human tumour cell lines have stable telomere settings, which are achieved by the negative regulation of the telomerase by the shelterin complex [25]. The shelterin complex consists of three shelterin subunits: *TRF1, TRF2* and *POT1*, which are interconnected by three additional proteins *TPP1, RAP1* and *TINF2*. *TINF2* (*TRF1*-interacting nuclear factor 2, or also called *TRF1*-Interacting Nuclear protein 2 (*TIN2*)) occupies a central position in the protein complex, by providing a bridge between the subcomponents of shelterin (for review see [26]). It has been shown that low expression of *TINF2* has a destabilising effect on shelterin [27], [28]. Due to the central and critical role of *TINF2*′ (*TIN2*) in shelterin stability we chose to measure the expression of this gene, as a proxy of shelterin activity.

The accumulation of shelterin along the telomeric repeat array prevents further telomerase induced telomere elongation [23]. Human cancer studies have shown that the joint up-regulation of telomerase expression and the genes of the shelterin complex may facilitate in stabilising the cancer cells by preventing the activation of DNA damage responses, such as apoptosis[27]. In approximately 85% of human cancers, telomerase activity has been shown to facilitate malignant transformation by maintaining replicative potential [29], [30]. In the remaining 10–15% of cancers, prevention of telomere loss is achieved in the absence of telomerase activity, through a recombination-mediated template switching mechanism known as alternative lengthening of telomeres (ALT) [31]. Consequently, tumour cells are able to escape apoptosis and hence maintain infinite replication capacity.

The DFTD cell line has been continuously dividing and adapting to the microenvironment of each different host for over 15 years. Therefore this clonally transmitted disease provides an unprecedented opportunity to study cancer cell evolution and progression *in vivo*. Increased knowledge of telomere homeostasis in this transmissible cancer may provide novel insights into how DFT cells achieve and maintain their hyperproliferative potential and may help us to understand the origins, somatic evolution and extraordinary success of this parasitic clonal lineage. Furthermore, both *TERT* and *TINF2* have been suggested as potential therapeutic targets in human cancer [32], [33] and increasing our understanding of the role of these genes in Devil Facial Tumour Disease may open novel avenues for disease treatment.

In the present study we focused on the role of telomere length, quantified as Telomere Copy Numbers (henceforth TCN) in hyperproliferation of DFT samples. As proxies for telomerase activity in tumour tissue samples we investigated the expression of the catalytic subunit of telomerase (*TERT*) and one of the main components of shelterin, the *TRF1*-intercating nuclear factor 2 (*TINF2*). Additionally, temporal changes in telomerase activity were quantified in five DFT cell lines.

## Results

### (a) Telomere Restriction Fragment length analyses

**Figure 1:**
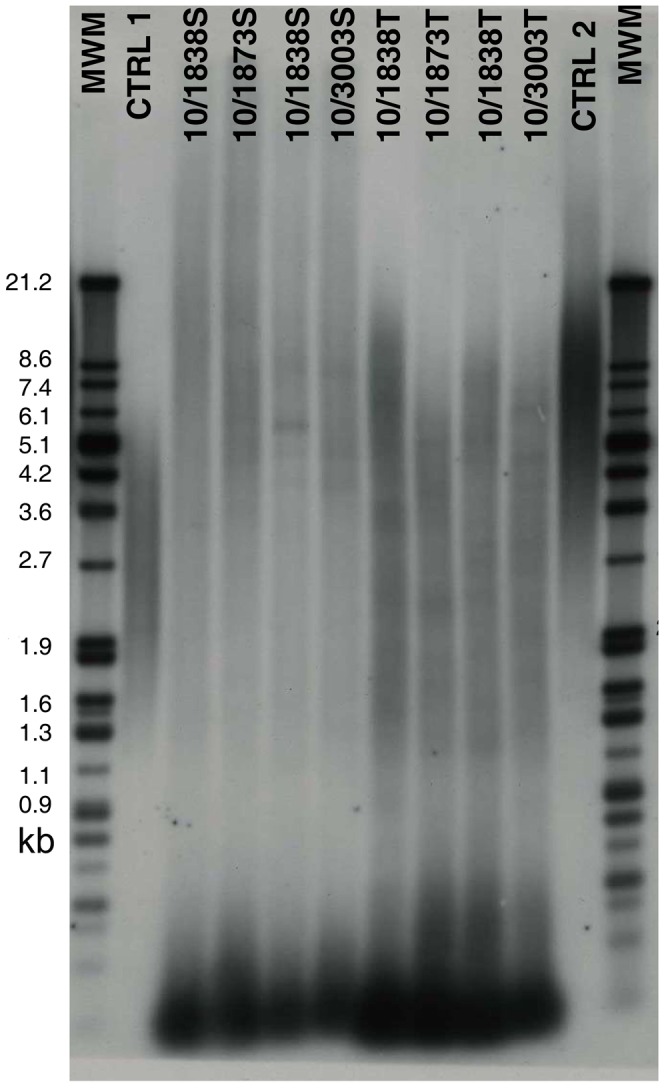
The Telomere Restriction Fragment Length analysis of devil samples revealed the presence of restriction enzyme recognition sites intercalated between the telomeric (TTAGGG)n sequences, preventing an accurate estimate of TRF lengths. Sample names depict the time of collection (10 = 2010). Identical numbers represent different tissue samples collected from the same animal, S depicts spleen and T depicts DFT samples. MWM stands for molecular weight markers. CTRL 1 indicates low molecular weight control DNA (length: 3.2 kbp), CTRL 2 indicates high molecular weight control DNA (length: 10.2 kbp). Control samples were supplied in the Telo TAGGG TL Assay Kit and originated from immortal cell lines.

Telomere restriction fragment (TRF) length was analysed in both primary and metastastatic tumours, as well as spleen samples from four Tasmanian devils (a total of 12 samples). We were unable to assign telomere length in all of the 12 samples as the restriction digest produced several repeated TRF-smears at various lengths (ranging from <2 kpbs up to <18 kpbs) (Figure 1).

### (b) Relative telomere repeat copy number

**Figure 2:**
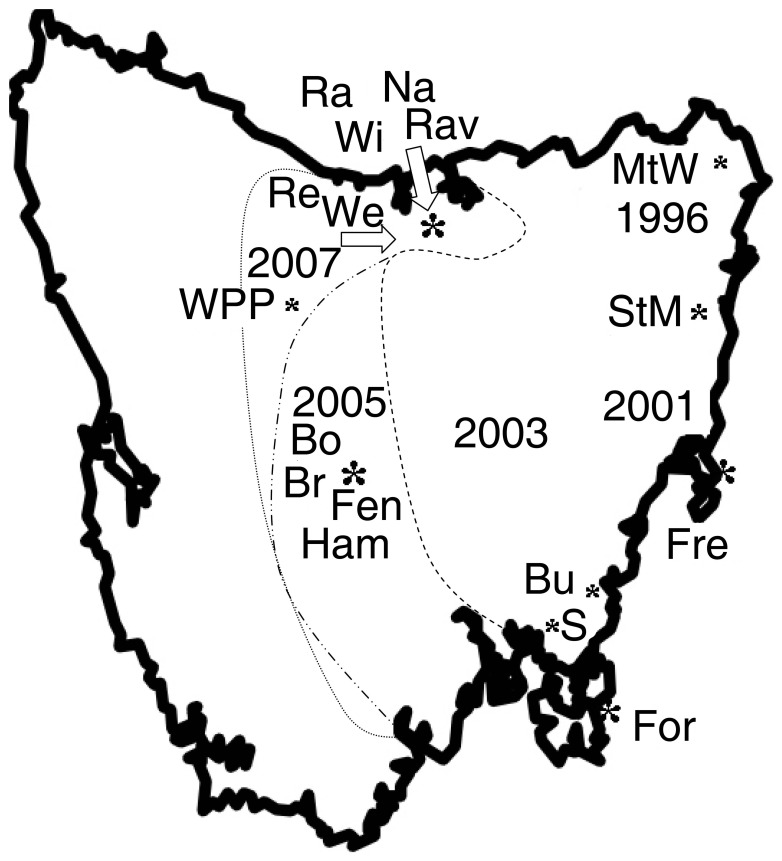
Tasmanian devil samples were collected from 17 locations across Tasmania. Tumour progression by 2003 depicted with dashed, by 2005 with dashed-dotted and by 2007 with dotted lines. Dates indicate the progression dates of DFTD. Location abbreviations: Bo = Bothwell, Br = Bronte Park, Bu = Buckland, Fen = Fentonbury, For = Forestier, Fre = Freycinet, Ham = Hamilton, MtW = Mt William, Na = Narawntapu, Ra = Railton, Rav = Ravenswood, Re = Reedy Marsh, S = Sorell, StM = St. Marys, We = Weegena, Wi = Wisedale, WPP = West Pencil Pine.

**Figure 3:**
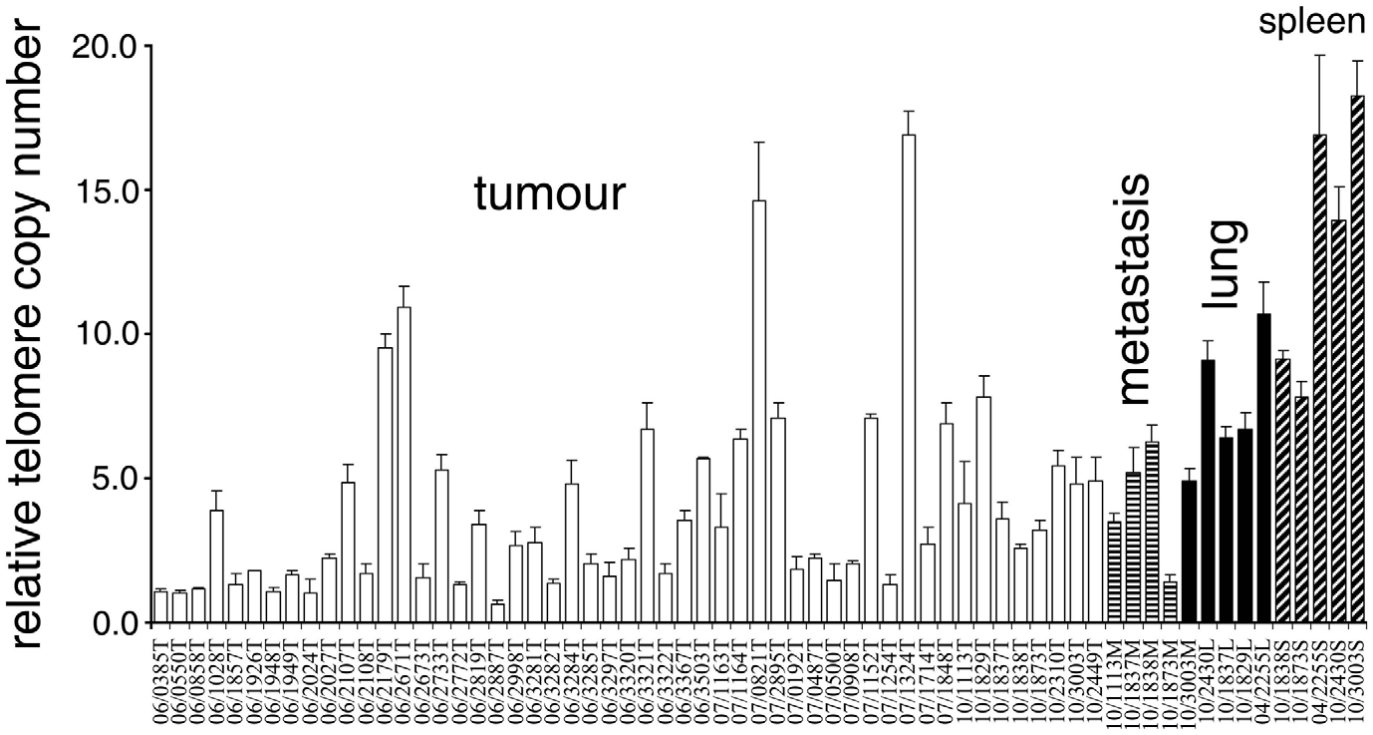
Relative telomere copy number is lower in DFT samples than in other devil tissues. Error bars depict standard deviations. Sample names indicate the time of collection (06 = 2006, 07 = 2007, 10 = 2010). Identical numbers represent different tissue samples collected from the same animal. Number of samples used in the analysis: primary tumours collected in 2006: N = 30, 2007: N = 13 and 2010: N = 8; metastasis: N = 4, spleen: N = 5, lung: N = 5.

Altogether 65 tissue samples, collected from 17 locations across Tasmania (Bothwell, Bronte Park, Buckland, Fentonbury, Forestier, Freycinet, Hamilton, Mt William, Narawntapu, Railton, Ravenswood, Reedy Marsh, Sorell, St. Marys, Weegena, Wisedale and West Pencil Pine, Figure 2), were included in the relative telomere repeat copy number analysis. Spleen, lung and tumour samples exhibited a significant TCN variation (Kruskal Wallis test: T = 17.1, P = 0.0007, DF = 3); with tumours (both primary and metastatic) showing the lowest (mean = 3.21±3.28) and spleen the highest (mean = 12.52±4.62), whereas lung samples showed intermediate TCN (mean = 8.03±2.04, Figure 3). A posthoc Conover-Inman test (available in StatsDirect) revealed no significant difference in TCN between lung and spleen (P = 0.52), between lung and metastasis (P = 0.08), between primary tumours and metastasis (P = 0.52). The same test did, however, reveal a significant difference in TCN between lung and primary tumours (P = 0.006), between spleen and tumour (P = 0.0001), and between spleen and metastasis (P = 0.01).

### (c) Temporal (longitudinal) geographical and strain variation in TCN

**Figure 4:**
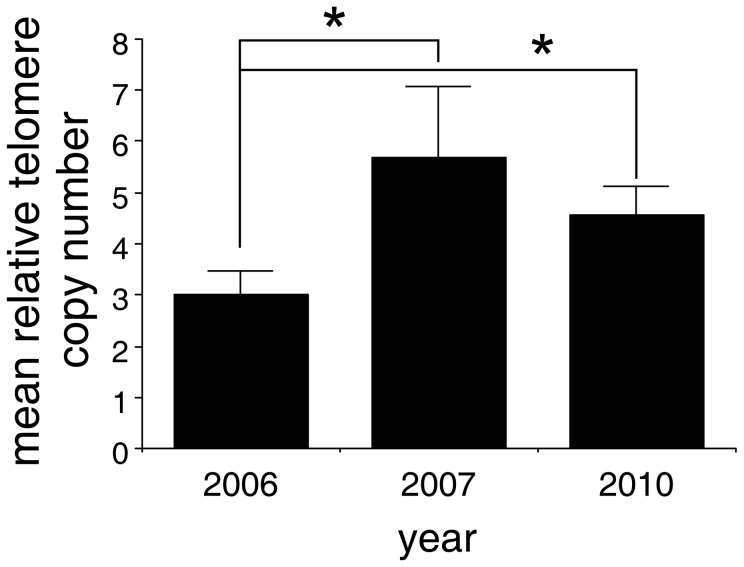
Relative mean DFT telomere copy number increases with time (2006: N = 30, 2007: N = 13 and 2010: N = 8). Error bars depict standard deviations. Significant differences in telomere copy numbers (TCN) were observed between years 2006 and 2007 (P = 0.03) and 2006 and 2010 (P = 0.01), but no significant difference in TCN was observed between years 2007 and 2010 (P = 0.6). * indicates significant differences.

A Kruskal-Wallis test revealed a significant temporal variation in TCN of samples collected in 2006, 2007 and 2010 (T = 7.66, P = 0.02, DF = 2) and a posthoc and a Conover-Inman test revealed significant differences in TCN between years 2006 and 2007 (P = 0.03) and 2006 and 2010 (P = 0.01), but no significant difference in TCN was observed between years 2007 and 2010 (P = 0.6). The post hoc test thus suggests a temporal increase in TCN (Figure 4).

We did not however, observe any significant difference in TCN among the three geographic regions (East, Central, North-West) (Kruskal Wallis test: T = 1.75, P = 0.42, DF = 2), nor among the four different strains (Kruskal Wallis test: T = 2.1, P = 0.56, DF = 3).

### (d) TERT and TINF2 expression

**Figure 5:**
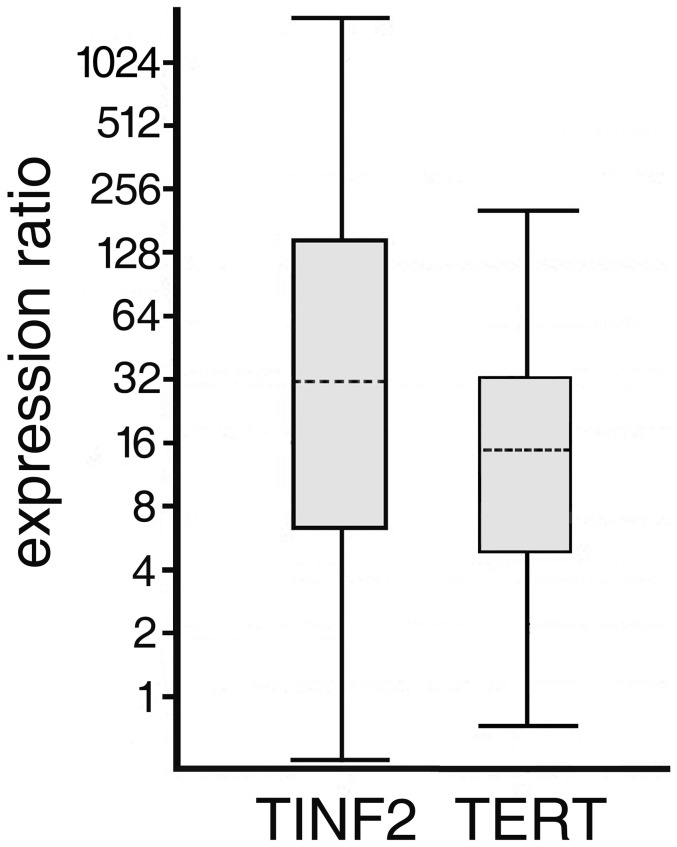
TERT and TINF2 genes are up-regulated in DFT samples compared to spleen (P<0.0001). Number of samples used in the analysis: tumours N = 11 and spleen N = 5. Stippled horizontal lines depict mean relative gene expression, boxplots indicate the range of standard error and bars depict 95% confidence intervals.

Devil *TERT* and *TINF2* expression was significantly up-regulated in primary tumours compared to spleen by a mean factor of 14.63 P<0.0001 (Std. Error (SE) ranging between 4.5 and 37.8), and 37.86 P<0.0001 (SE ranging between 4.6–272.9), respectively (Figure 5). *TERT* and *TINF2* expression showed substantial variation across tumour samples. We did not, however, observe any significant association between TCN and *TERT* or *TINF2* expression in the tumour samples (Spearman rank correlation, R = −0.31, P = 0.36, N = 11; R = −0.05, P = 0.88, N = 11, respectively).

**Figure 6:**
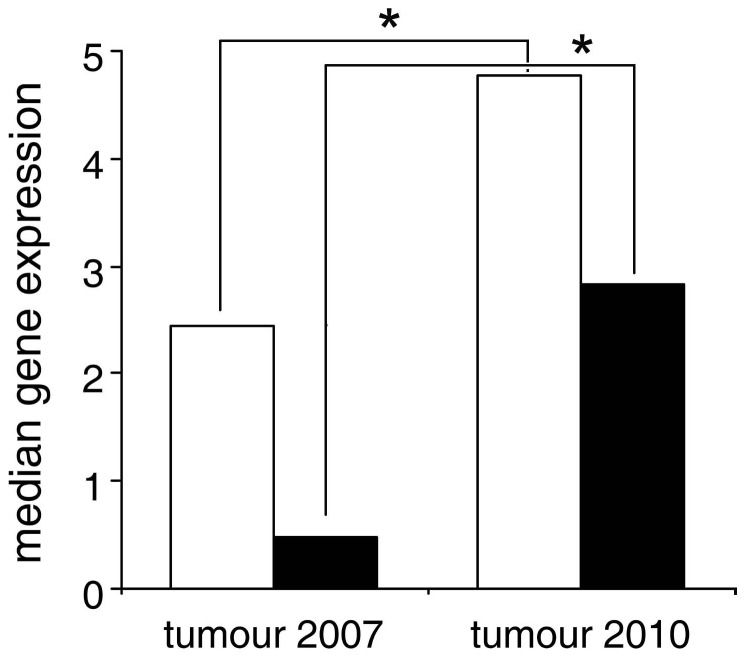
TERT and TINF2 gene expression increases with time in DFT samples. Open bars depict TERT expression, black bars depict TINF2 expression. Number of samples used in the analysis: tumours collected in 2007: N = 5 and 2010: N = 6. * depicts significant differences (P = 0.0173 and P = 0.013, respectively).

In the tumours, the expression level of *TINF2* was significantly lower than that of *TERT* (Two-sided Mann-Whitney U-test; U = 27, P = 0.03, median *TINF2* = 0.51, median *TERT* = 3.58). Despite the difference in expression level we observed a significant association between *TERT* and *TINF2* (Spearman rank correlation R = 0.83, P = 0.0017, N = 11). We also detected a significant temporal variation in *TERT* and *TINF2* expression levels as both *TERT* and *TINF2* showed significantly increased expression levels in 2010 compared to 2007 (Two sided Mann-Whitney U-test, U = 28, P = 0.0173 and U = 28.5, P = 0.013, respectively; *TERT* median 4.77 and 2.44, respectively; *TINF2* median 2.84 and 0.47, respectively, Figure 6).

### (e) Telomerase activity assay

**Figure 7:**
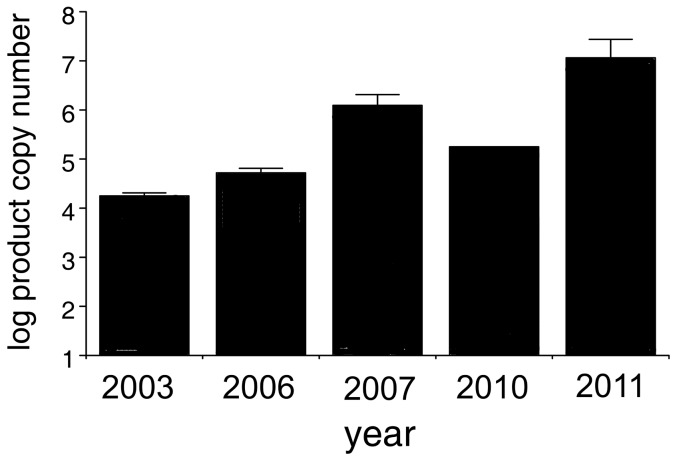
Telomerase activity increases over time in DFT cell lines (P = 0.037). The five cell lines originated from tumour samples collected in 2003, 2006, 2007, 2010 and 2011. Bars represent standard errors between technical repeats (N = 3).

Telomerase activity was detected in five of the DFT cell line samples. Moreover, a positive temporal shift was observed in telomerase activity (total product generated) from 2003 to 2011 (Spearman rank correlation, R = −0.9, P = 0.037, N = 5, Figure 7).

## Discussion

Like many human cancers [34], [35], Devil Facial Tumours have short telomeres. *TERT* gene expression is up-regulated 15-fold in DFT cells compared to spleen cells, and telomerase activity is present in DFT cell lines. This telomerase activity prevents DFT cells from entering replicative senescence, and taken together with the lack of extrachromosomal telomeric DNA (Pearse pers. com) in DFT karyotypes, points towards telomerase up-regulation, not alternative lengthening of telomeres (ALT), as the primary mechanism for DFT immortality [31].

High expression of *TINF2*, a negative regulator of telomerase activity [23], in DFT cells suggests that telomere elongation is highly regulated in this cancer. DFT cells appear to monitor and regulate the length of individual telomeres i.e. shorter telomeres are elongated by telomerase activity; longer telomeres are protected from further elongation by the shelterin complex [27].

Increased *TERT* and *TINF2* expression most likely explain the lack of spatial variation in DFT TCN. Also, TCN does not differ between DFTD strains. However, the temporal increase in *TERT* and *TINF2* gene expression and enzyme activity may be linked with growth advantage and increased tumour progression in DFT, as it is in human cancers [34], [36], [37].

The short telomeres and up-regulation of telomerase likely counteract each other. The short telomeres lead to increased genetic instability but the telomerase activation facilitates tumour growth by either inhibiting further chromosomal instabilities or by circumventing checkpoints that recognise dysfunctional telomeres [37]. Longer telomere lengths may ensure the success and survival of DFT cells by stabilising chromosomal rearrangements and preventing further genomic instabilities.

DFT samples exhibited consistently lower TCN compared to other tissues, but a considerable (16-fold) among-sample variation of TCN was observed. Methodological challenges, most likely caused by interruption of telomeric repeats with restriction sites (a common feature of marsupial chromosomes) [38], prevented us from correlating variation in copy number to variation in telomere length. *TERT* and *TINF2* RNA levels did not associate with TCN, a finding also observed in other human cancer cell lines [39].

Future research should investigate the impact of telomere length variation on tumour fitness. Is there an evolutionary optimum around telomere length? Do selective forces maintain telomere length equilibrium or select for tumour cells with longer telomeres? Will increased *TERT*, *TINF2* gene expression, telomerase activity and longer telomeres lead to the development of a more stable DFT form with higher growth and proliferative potential?

Since its first appearance 15 years ago, DFTD has been passed through thousands of devils, killing close to 80% of the animals, without undergoing replicative senescence. DFTD thus represents one of the oldest naturally living, and continuously passaged cell lines in nature. We have shown that the dynamic interaction between the telomerase and shelterin complex is essential to the success of this parasitic, transmissible cancer. Selection has promoted the progression of DFT cells with increased telomere copy numbers as well as increased gene expression and telomerase activity, both of which may ultimately lead to a faster growing tumour. DFTD provides a powerful model system to understand tumourigenesis not only in wildlife but also in human cancers. Further studies focusing on understanding the exact mechanism of telomere maintenance and regulation in DFT cells will lead to better understanding of the evolutionary strategies and mechanisms underlying and maintaining the unlimited proliferative potential of cancer cells.

## Materials and Methods

### (a) Samples

Tissue samples were collected from 17 locations across Tasmania (Bothwell, Bronte Park, Buckland, Fentonbury, Forestier, Freycinet, Hamilton, Mt William, Narawntapu, Railton, Ravenswood, Reedy Marsh, Sorell, St. Marys, Weegena, Wisedale and West Pencil Pine, Figure 1) by the members of the Department of Primary Industries, Parks, Water and Environment (DPIPWE) from euthanised DFTD affected devils and stored at the Animal Health Laboratories (DPIPWE). The research was carried out with approval from the DPIPWE (Department of Primary Industries, Parks, Water and Environment, Tasmania) animal ethics comitte, Animal Ethics No: 101 2010/11. We obtained tissue samples from 48 devils. We had access to spleen and primary tumour, as well as metastasis samples from four devils. 51 primary tumours, five metastases, four lung and five spleen DNA samples were used in the analyses (Figure 2).

In order to investigate geographical/spatial variation in telomere copy number, the samples were divided into three geographical regions (East = distribution prior to 2003, Central = distribution between 2003–2005 and North-West = distribution between 2005–2007) based on the temporal/spatial progression of DFTD across Tasmania [40]. We had information about the specific strains of 45 tumour samples and used these samples to investigate telomere copy number variation between DFT strains.

Temporal variation in telomere attrition rates was based on copy numbers observed in samples collected in 2006, 2007, and 2010 (N = 51). Due to sample collection procedures we were only able to extract RNA from five spleen and 11 (5 from 2007 and 6 from 2010) primary tumour samples which were subsequently analysed with the Quantitative Real-Time PCR.

The telomerase activity assays were performed on five lysed DFT cell line samples obtained from DPIPWE. Tumour cell lines were generated and maintained at DPIPWE according to the descriptions of Pearse et al 2012 [11]. The five cell lines originated from tumour samples collected in 2003, 2006, 2007, 2010 and 2011.

### (b) DNA extraction, telomere fragment length measurements and telomere copy number quantitation

Genomic DNA was isolated from tissue samples by phenol-chloroform extraction. Sample DNA quantity and quality was measured using a NanoDrop Spectrophotometer (NanoDrop Technologies, Wilmington, DE).

### (c) Telomere Restriction Fragment Length analyses (TRFL)

Telomere restriction fragment (TRF) length was quantified by Southern blot hybridisation following the protocol outlined in the Telo TAGGG TL Assay Kit (Roche, Indianapolis, IN), using constant field electrophoresis. After digesting genomic DNA with a mixture of *Hinf*I and *Rsa*I restriction enzymes to remove sequence-diverse DNA from the centromeric side of the telomeres, telomere length was analysed on agarose gels, which were run for 4 hours at 5 V/cm. The TRF fragments were labeled with a digoxigen labeled probe, and the TRF images were subsequently developed on high performance chemiluminescence film (Amersham Bioscience, Waukesha, WI).

### (d) Quantitative PCR of relative telomere copy number

Relative telomere repeat copy number was analysed by quantitative PCR (Q-PCR) as described by Cawthon [41]. Telomere specific primers were adopted from Cawthon [41], Tel1: 5′- GGTTTTTGAGGGTGAGGGTGAGGGTGAGGGTGAGGGT -3′; Tel2, 5′-TCCCGACTATCCCTATCCCTATCCCTATCCCTATCCCTA -3′. *RPLP0* (also called *36B4*) gene was chosen as single copy gene following the methodology of Cawthon [41], and the single copy presence of this gene in the Tasmanian Devil genome [42] was confirmed by searching the genome for alternative copies of the gene. No alternative *RPLP0* copies or pseudogenes were found. *RPLP0* gene specific primers were designed based on the Tasmanian Devil genome sequence [42], using the Primer3Plus website (http://www.bioinformatics.nl/cgi-bin/primer3plus/primer3plus.cgi), *RPLP0_F*: 5′- CTTCCCGTTCACCAAAGAAG -3′ and *RPLP0_R*: 5′- TGTTCTGGACTGGCAAAGTG -3′. The Q-PCR reactions were performed on the RotorGene6000 (Qiagen, Germantown, MD) in 15 µl total volume, containing 7.5 µl of Qiagen 2xQuantifast Sybr Green PCR master mix (Qiagen, Germantown, MD), 0.5 µM forward and reverse primers (the optimal primer concentrations) and 1 µl of gDNA (1 ng/µl concentration). Q-PCR conditions were established according to the manufacturer protocol: 95°C for 5 min denaturation followed by 40 cycles of 95°C for 15 s and 60°C for 30 s (annealing temperature). Fluorescence signal was acquired at the annealing temperature. Standard curves were generated using serial 1∶5 (*RPLP0*) and 1∶10 (Telomere) dilutions of a composite sample containing equal parts of DNA from spleen and tumour tissue extracts. All dilutions were run in triplicate. Standard curves had an R^2^>0.985 (*RPLP0* reaction: R^2^ = 0.985 and Telomere reaction R^2^ = 0.997) and contained at least four (Telomere reaction) or five (*RPLP0* reaction) dilutions from the dilution series with a linear dynamic range of at least 3 orders of magnitude and had PCR efficiencies between 1.3 and 1.1 (respectively). All samples were run in quadruplicate, and all Cq values for unknowns fell within the linear quantifiable range of the appropriate standard curves. The program Rest [43] was used to calculate the normalised fold change of the target gene compared to the reference gene. This program package also corrects for different reaction efficiencies. Statistical significance (P<0.05) was determined by a Pair Wise Fixed Reallocation Randomisation Test© as described by Pfaffl *et al .* [43].

### (e) RNA extraction and quantifying telomerase expression by quantitative RT-PCR

RNA was extracted from tissue samples using a combination of Trizol (Sigma, St. Louis, MO) and Qiagen RNeasy mini kit (Qiagen, Germantown, MD). RNA quality and quantity were quantified on an Agilent 2100 BioAnalyzer (Agilent, Santa Clara, CA). Genomic DNA was removed from the RNA samples by the DNAse I AMPD1 kit (Sigma, St. Louis, MO) and cDNA was synthesized with the QuantiTect Reverse Transcription Kit (Qiagen, Germantown, MD). Telomerase gene specific primers spanning exon boundaries were designed in the catalytic protein subunit of devil *TERT* gene, using the Primer3Plus website (http://www.bioinformatics.nl/cgi-bin/primer3plus/primer3plus.cgi) *TERT*-F: 5′- TGCTGTAGTCCAGAAGAATGC -3′, and *TERT*-R: 5′- TGCAGGGAAGAGGTTTCTTG -3′. *TRF1*-interacting nuclear factor 2 (*TINF2*) primers were designed across exons 6 and 7 *TINF2*-F: 5′- TTGCCCTGACTCAGTATTGC -3′, and *TINF2*-R: 5′- GGATCCTGGAAAACTTGCTC -3′. Two genes, *GAPDH* (*qGAPDH*) and *GUSB* (*qGUSB*) were used as normaliser genes following the description of Murchison et al.[10], [14]: *qGAPDHf*: 5′- GACTCAACCACGTATTCGGCTC -3′and *qGAPDHr*: 5′- ATATGATTCCACCCATGGCAAGTTCAA -3′; *qGUSBf*: 5′- CTGCTGCCTATTATTTCAAGAC -3′and *qGUSBr*: 5′-CAAGATCCAATTCAGGCTTAG -3′. The Q-PCR reactions were performed on the RotorGene6000 (Qiagen, Germantown, MD) in 15 µl total volume, containing 7.5 µl of Qiagen 2xQuantifast Sybr Green PCR master mix (Qiagen, Germantown, MD), 0.5 µM forward and reverse primers (the optimal primer concentrations) and 1 µl of cDNA (5 ng/µl concentration). Reverse transcriptase negative and cDNA negative samples were run alongside the cDNA samples as controls to detect genomic DNA contamination and primer-dimer formations. Q-PCR conditions were established according to the manufacturer protocol: 95°C for 5 min denaturation followed by 40 cycles of 95°C for 15 s and 60°C for 30 s (annealing temperature, AT). Fluorescence signal was acquired at the AT. To evaluate the specific amplification a final melting curve analysis (from AT up to 99°C) was added under continuous fluorescence measurements. Standard curves were generated using serial 1∶5 dilutions of a composite sample containing equal parts of cDNA samples generated from spleen and tumour tissue RNA extracts. All dilutions were run in triplicate. Standard curves had an R^2^>0.99 (*GUSB* reaction: R^2^ = 0.998, *TERT* reaction: R^2^ = 0.990 and *TINF2* reaction: R^2^ = 0.993) and contained at least four (*TERT* reaction) or five (*GUSB* and *TINF2* reactions) dilutions from the dilution series with a linear dynamic range of at least 3 orders of magnitude and had PCR efficiencies between 0.98 and 1.4 (*GUSB*: 1.1, *TERT*: 1.4 and *TINF2*: 0.98). All samples were run in quadruplicate, and all Cq values for unknowns fell within the linear quantifiable range of the appropriate standard curves. The program Rest [43] was used to calculate the normalized fold change of the target gene compared to the reference gene. This program package also corrects for the different reaction efficiencies. Statistical significance (P<0.05) was determined by a Pair Wise Fixed Reallocation Randomisation Test© as described by Pfaffl *et al.* [43].

### (f) Telomerase assay

Telomerase activity was measured using the TRAPEZE-RT telomerase detection kit (Millipore, Bedford, MA). Cells were lysed in 200 µl of CHAPS buffer, and protein content was quantified by using the Pierce BCA Protein Assay Kit (Thermo Scientific, Rockford, IL). Aliquots of cell lysate (250 ng of protein/well) were assayed in triplicate. Standards, inactivated samples, and non-template reactions were also included in the assay as quality controls. Real-time amplifications were performed with a RotorGene6000 multicolor real-time PCR detection system (Qiagen, Germantown, MD). Standard curve was generated from TSR8 control template following the manufacturer's instructions. Telomerase activity (total product generated) was calculated by extrapolating the average Ct values from the sample wells to the standard curve.

### (g) Statistical analyses

Relative quantifications of telomere copy numbers and gene expression were performed using sample crossing points, and data was analysed with the RotorGene6000 software 1.7. (Qiagen, Germantown, MD), applying the “second derivative maximum” method [44]. The Excel application Best-Keeper [43] was used to check the data for statistical significance, normality and reliability, and the normaliser gene *GUSB* was chosen as reference based on BestKeeper calculations [45]. The program Rest [43] was used to calculate the normalised fold change of the target gene compared to the reference gene. Statistical significance (P<0.05) was determined by a Pair Wise Fixed Reallocation Randomisation Test© as described by Pfaffl *et al.* [43]. When data could not be transformed to achieve normality non-parametric statistics were applied using the software packages StatsDirect and JMPv5 [46], [47].

## Acknowledgments

We are grateful to Kate Swift, Pamela Hodson and Bobby Hua for providing the lysed tumour cell line samples. We thank the Save the Tasmanian Devil Program and researchers from the School of Zoology at the University of Tasmania for collecting samples. We are grateful to Dr. Gabriele Saretzki and two anonymous reviewers for their comments, which have led to substantial improvements to this manuscript. KB is supported by an ARC Future Fellowship. ATP is supported by an NHMRC Career Development Fellowship. ATP′s contribution was made possible through Victorian State Government Operational Infrastructure Support and NHMRC IRIISS.
